# Cleaning Efficacy and Debris Extrusion using XP-Endo Finisher and XP-Endo Finisher R as Supplementary Files during Retreatment: An *in Vitro* Study

**DOI:** 10.14744/eej.2021.44366

**Published:** 2022-03-11

**Authors:** Ekramy HASSAN, Marwa SHARAAN, Mai RAGAB

**Affiliations:** From the Department of Endodontics (E.H., M.S. 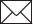 marwaelsayedsharaan@gmail.com, M.R.), College of Dentistry, Suez Canal University, Egypt

**Keywords:** Debris extrusion, D-Race, retreatment, XP-Endo Finisher, XP-Endo Finisher R

## Abstract

**Objective:**

The aim of the study was to examine the efficacy and debris extrusion of XP -Endo Finisher and XP-Endo Finisher R when used in the removal of root canal filling as supplementary files.

**Methods:**

Sixty single-rooted mandibular premolars with single canals were selected. After root canal preparation and obturation, roots were distributed across four groups according to the method of retreatment (n=15): H files, D Race files, D Race +XP-Endo Finisher and D Race+ XP -Endo Finisher R. After retreatment completion, the debris was dried in a hot air oven and weighed. Later, the coronal, middle and apical thirds were assessed using the stereomicroscope. One-way ANOVA followed by Tukey’s post hoc test was used to compare across all tested groups. The significance level was set at 0.05 (P<0.05).

**Results:**

XP- Endo Finisher R exhibited significantly cleaner root canals than XP- Endo Finisher (41.58±10.56 and 52.68±9.94 respectively) and extruded more debris apically (16.56%±4.07 and 12.82%±3.41 respectively) (P<0.001).

**Conclusion:**

Although none of the tested approaches rendered root canals free of filling remnants, the XP-Endo Finisher R cleaned canals significantly more than the XP-Endo Finisher and extruded more debris apically.

**HIGHLIGHTS**

• No tested retreatment technique could completely clean root canals from failed filling materials.
• Every investigated retreatment technique extrudes debris apically to some degree.
• H files were the least effective in cleaning root canals and extruded the greatest amount of debris apically during retreatment.
• D-Race files were the fastest technique in retreatment among the groups present in this study and extruded the least amount of debris apically.
• XP- Endo Finisher and XP- Endo Finisher R significantly increased the cleaning ability when used as supplementary approaches, but XP- Endo Finisher R was more effective and extruded more debris apically.

## INTRODUCTION

Root canal retreatment procedures are often challenging that require complete removal of root canal filling materials. Proper mechanical preparation is crucial to regain access to the apical foramen. This enables irrigants to reach and disinfect the root canal space before sealing all portals of exits (1).

Recently, a new instrument, XP-Endo Finisher file (FKG Dentaire, La Chaux-de- Fonds, Switzerland) was introduced to the market. It is a # 25 tip non-tapered rotary Nickel Titanium (NiTi) instrument made of a special alloy (MaxWire; Martensite-Austenite Electropolish Flex) (2). XP-Endo Finisher file is straight in its martensitic phase, which is achieved below 30°C. However, when placed in the canal at the body temperature, it changes to the austenitic phase assuming a spoon shape in the last 10 mm with a depth of approximately 1.5 mm (2). When rotating, this instrument achieves a natural diameter of 3 mm in the last 10 mm (2). The manufacture claims that when the instrument tip is squeezed, the bulb expands to 6 mm; when the bulb is compressed, the tip expands to 6 mm. The natural constrictions and expansions in the canal will alternatively cause the bulb and tip to expand and contract when the XP-Endo instrument is pushed up and down for 7 to 8 mm inside the canal (2). This pattern causes the instrument to scrape the canal walls prompting the turbulence of the irrigation solutions (3).

The XP-Endo Finisher R (FKG Dentaire, La Chaux-de- Fonds, Switzerland) is a new variant of the XP-endo Finisher file. It is designed for retreatment cases, according to the manufacturer, and the file has a bigger core diameter (tip # 30), and changes in the instrument tip, making it stiffer and thus more aggressive. The goal of these adjustments is to improve its ability to touch and remove root filling components that remain after traditional retreatment procedures (4). A study conducted on maxillary canines obturated with zinc oxide eugenol sealer with a thermo-mechanical compaction technique revealed that there is no significant difference between the retreatment efficacy of S1 reciprocating file and D-Race. However, using XP-Shaper as a supplementary approach improved cleaning results (5). A cone beam computerized tomographic evaluation revealed promising results in cleaning efficacy and root dentine preservation when XP- Endo Finisher R and XP-Shaper were used; these files were more effective than the XP- Endo Finisher file (6).

Further challenges can occur during the root canal retreatment including the apical extrusion of dentine chips, gutta-percha, remaining pulp tissues, and microorganisms (7). These extruded products are associated with flare ups, post- operative pain or endodontic failure (8). Extruded debris resulting from retreatment can exceed initial treatment debris (9). A recent study, using mandibular premolars obturated by warm vertical compaction technique with AH plus sealer, revealed that XP Shaper, during retreatment, extruded less debris apically than Reciproc blue (VDW, Munich, Germany) (10).

To date, no published research has investigated the quantity of the apical extruded debris during an endodontic retreatment procedure using XP-Endo Finisher and XP-Endo Finisher R as supplementary files. Our null hypothesis was that there is no significant difference between the tested techniques in cleaning efficacy and apical debris extrusion during retreatment procedures.

## MATERIALS AND METHODS

The present study was started after the approval of the Research Ethics Committee at the College of Dentistry, Suez Canal University (registration no. 216/2019).

### Sample size calculation

Based on previous studies, sample size calculation was performed using G*Power version 3.1.9.2. The effect size was 0.57 using an alpha (α) level of 0.05 and a beta (β) level of 0.05, i.e., power=95%; the estimated minimum sample size (n) was 60 teeth for the four groups in this study, 15 teeth per group (6, 10).

### Sample collection

Sixty human mandibular premolars were only used to standardize the canal morphology. They were freshly extracted for periodontal or orthodontic purposes. After viewing the teeth using bidirectional radiographic images displaying buccolingual and mesiodistal directions. The study only included teeth having a single, straight root canal (10°) according to the Schneider method (11). Teeth of comparable dimensions with an initial apical diameter equal to a # 15 K file (Mani Inc., Tochigi, Japan) were selected. 

The following inclusion criteria were applied to the samples: 1) free from root caries, 2) single canals type I Vertucci's classification with mature apex, 3) no internal resorption or calcification, 4) no previous endodontic treatment, and 5) and no signs of cracks.

Teeth were immersed in 2.5% sodium hypochlorite for 2 hours to enable disinfection (12).

The remaining external tissue fragments and calculus were removed from the external surface of teeth by ultrasonic scaling. Teeth were then kept in saline until use. All samples were decoronated using a high speed diamond bur and copious amount of coolant (13). Decoronation was completed for all selected teeth enabling straight line access, for standardization purposes, at fixed length measured from tooth apex to be 16 mm.

### Root canal treatment

Roots were placed in wet gauze to avoid dehydration. K file # 10 (Mani Inc., Utsunomiya, Tochigi, Japan) was introduced to the root canal and moved down gently until it was seen from the major apical foramen. Then, subtraction of 1 mm to obtain the working length (WL). Samples were prepared with Bio-Race instruments (FKG Dentaire de Chaux Fonds, Switzerland) BR0 25/ 0.08, BR1 15/ 0.05, BR2 25 /0.04, BR3 25 /0.06 and BR4 35/ 0.04 via gentle pecking motion to the WL. The endodontic motor (VDW GmbH, Munich,Germany) was adjusted according to the Bio-Race manufacturer’s instructions at a speed of 600 rpm and torque of 1 Ncm. Flute cleaning was completed after each file removal from the root canal (14).

At each instrument, the canals were irrigated with 1 mL 2.5% NaOCl (Meta Biomed CO.LTD.,Chungbuk.Korea) using a NaviTip 30-G needle (Ultradent, South Jordan, USA). The needle was 2 mm away from the WL. After root canal preparation, the samples were irrigated using 17% EDTA (Meta Biomed CO.LTD., Chungbuk, Korea) activated for one minute. IrriSafe instrument (Satelec, Acteon, Merignac, Aquitaine, France) 25/21 mm was mounted onto an ultrasonic unit (NSK, Nakanishi, Tochigi, Japan) for passive ultrasonic irrigation. Later, rinsing was performed with 2.5% NaOCl, and then distilled water (15).

Lateral compaction was utilized in obturation after dryness of the root canal with 35/ 0.04 paper point (Meta Biomed CO.LTD., Chungbuk, Korea). AH plus sealer (Dentsply, Konstanz, Germany), was mixed according to the manufacturer’s instructions. The tip of Gutta-Percha cone # 35/0.04 (Meta Biomed CO.LTD, Chungbuk, Korea) was lightly coated with AH plus and gently introduced into the canal. Then finger spreader, # 25/ 0.02 (Mani Inc., Utsunomiya, Tochigi, Japan), was inserted in the canal rotated and withdrawn. Accessory Gutta-Percha cones # 25/ 0.02 coated with thin layer of AH plus were placed into the canal until there was no more space for accessory cones beyond 2-3 mm in the canal. After root canal filling was completed, a periapical radiographic image was taken for evaluation (16).

After root canal treatment completion, access cavity was sealed by Coltosol F temporary filling (Coltene/Whaledent Inc USA). Teeth were stored at 37°C in a 100% humidity incubator (Jinan, Shandong, China) for 7 days (15).

### Randomization of samples

One of the co-authors, who was not involved in the steps of retreatment, performed the blind allocation of the teeth. This allocation was completed via Microsoft Excel randomization completed in groups. Roots were equally divided and randomly placed into 4 groups according to the instruments used in retreatment (n=15).

### Retreatment procedure

#### Group 1 (G.G +H)

Root canal filling in the coronal and middle thirds was removed using Gates-Glidden burs (Mani Inc., Utsunomia, Tochigi, Japan) # 4 and 3 in combination with hand H files via the crown down technique. The filling mass was penetrated by # 60 H file followed by 55, 50, 45 and 40 until the WL was regained with file # 35. The apical third was enlarged to a # 40 K hand file (16).

#### Group 2 (D-Race)

Retreatment was performed using D-Race (FKG Dentaire, La Chaux-de- Fonds, Switzerland). The coronal third and beginning of the middle third of the root filling were removed using the DR1 instrument 30/0.10 at a speed of 1000 rpm and a torque of 1.5 Ncm. The DR2 instrument 25/ 0.04 was used at a speed of 600 rpm and a torque of 1 Ncm until the working length was maintained. Apical preparation was attained by Bio-Race instruments BR3 25/ 0.06, BR4 35/ 0.04 and BR5 40/ 0.04 at a speed of 600 rpm and a torque of 1 Ncm.

For both groups 1and 2, to keep patency of the canal, K file #10 was used to confirm canal path before reintroducing the next file. Moreover, after each instrument, 2.5% NaOCl with 1ml volume was utilized. Upon withdrawal, the files were cleaned of any obturating material before being reintroduced into the root canal. Retreatment procedures were continued until no gutta-percha remnants were observed on the files.

#### Group 3 (D-Race + XP-Endo finisher)

Retreatment was performed with D-Race files as described in group 2. In addition, XP–Endo Finisher file was used as a supplementary file following the retreatment procedures. The XP-Endo Finisher file was used according to the manufacturer’s instructions at a speed of 800 rpm and a torque of 1 Ncm. The instrument was placed in a contra- angle hand piece and was removed from the plastic tube in rotation mode by applying a lateral movement. With no rotation, the XP-Endo Finisher file was placed into the canal. Subsequently, rotation was started, and the instrument was activated for 1 min using slow and gentle 7-8 mm lengthwise movements up to the WL.

The instrument was used in a brushing action against the root canal walls. All procedures with the XP-Endo Finisher files were performed at 37°C inside an incubator. Irrigation was performed using 2.5% NaOCl with 1ml volume with each instrument during the procedures. The irrigation solutions were also warmed and kept at 37°C in a water bath. This was done to simulate the body temperature and create appropriate conditions for the XP-Endo Finisher instruments to undergo the phase change.

#### Group 4 (D-Race + XP-Endo finisher R)

Retreatment was completed with D-Race files as described in group 2 and then as in group 3. XP-Endo Finisher R file was used as supplementary file following the retreatment procedures. 

### Methods of evaluation

Empty vials with holes in their stoppers were weighed with an electronic balance (Sartorius Entris 64i-1S, Sartorius, Massachusetts, USA) with an accuracy of 10^-4^ g (8). After calibration for the scales was performed, each vial was placed on the scale for three times. Then, the mean weight of each vial was verified. Separation of the Eppendorf tubes stoppers was completed, followed by drilling a hole on the tops. Each root sample was placed up to the cementoenamel junction at the stoppers of the Eppendorf tubes. A 27-G needle was inserted next to the stopper (to equalize the internal and external pressures). Any gaps near the hole, the Eppendorf tube, and the needle were sealed with an adhesive to stop any irrigant leakage around the hole. The Eppendorf tube stopper including the tooth, was then joined to the vial (17).

**Figure 1.**
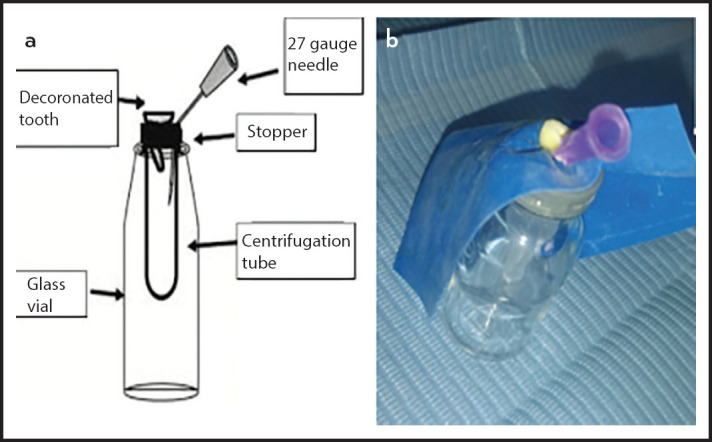
(a) Diagram and (b) Photograph of the root mounted into the vial with needle vented (technique for debris collection)

Throughout the retreatment procedure, the tooth was isolated with a rubber dam to stop the operator from seeing the root and to avoid irrigant seepage in the hole as shown in Figure 1. For irrigant suction, an aspirator was used which overflowed from the top of root sample. When the retreatment procedure was completed, the stoppers of vials and Eppendorf tubes were removed. The surface of the root was washed with 1 ml distilled water; this water was placed into the vial to collect the debris adhering to the root surface. The vials were stored in a hot air oven at 50°C for 10 days to evaporate the distilled water before weighing the dry debris.

The debris dried in a hot air oven were weighed three times with an electronic balance that has a 10^-4^ accuracy (18). Then the mean weight for each sample was recorded. The mass of the apical extruded debris was obtained by subtracting the initial weight of the vials from the vials’ final weight.

**Figure 2.**
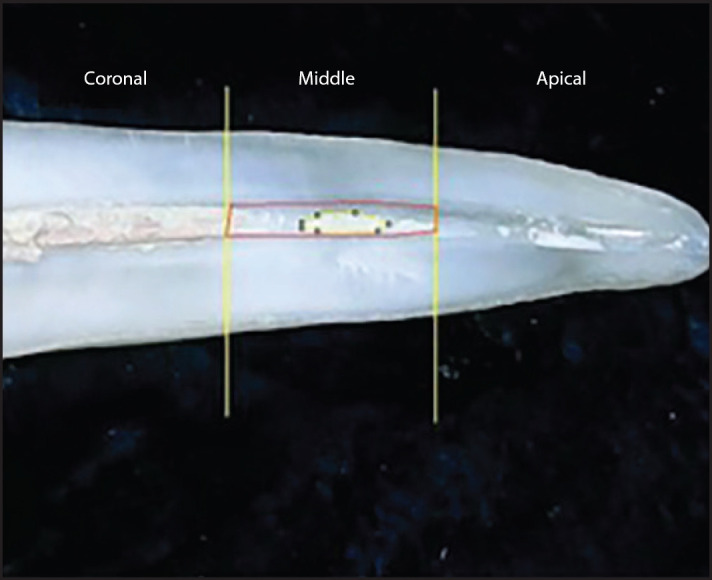
Photograph showing image analysis by Image J software displaying area selection and the analysis of remnants of filling materials in the coronal, middle and apical thirds of the root

Two shallow longitudinal grooves were made in the buccolingual direction of the roots with care, to avoid penetration of the root canals. Each root sample was then split longitudinally into mesial and distal halves using chisel; for each specimen, the half with the most intact root canal was used (19). Afterwards, the longitudinally sectioned roots were evaluated. Coronal, middle and apical thirds were assessed using the stereomicroscope at 30x magnification and photographed with digital camera (Nikon MA100 Nikon, Minato-ku, Tokyo. Japan). Image J software (Java-based image processing program developed at the National Institutes of Health and the Laboratory for Optical and Computational Instrumentation (LOCI), University of Wisconsin) (20) was used to investigate the surface area of the root canal third and the remnants of root canal filling materials as displayed in Figure 2. The total area of each third, and areas covered by remaining root canal filling materials (RCF) in each third were determined. Then, areas covered by remaining RCF were divided by the total area of each third multiplied by 100 to obtain the percentage of remnants of RCF for each third. The data was calculated after blindly taking the average of three readings by the three examiners.

Using a stopwatch, the time of retreatment via different techniques was evaluated by calculating the time from when the first instrument introduced into the root canal until the last instrument was used, subtracting the time needed for irrigation and instruments changing (21).

### Statistical analysis

Statistical analysis was carried out using the SPSS software (IBM Corp, USA Statistics Version 20 for Windows) at P≤0.05 level of significance. The Kolmogorov-Smirnov and Shapiro-Wilk tests showed that data were normally distributed. Therefore, One-way ANOVA followed by Tukey’s post hoc tests and the Paired sample t-test were used to compare between different groups.

## RESULTS

### Cleaning efficacy

**Figure 3.**
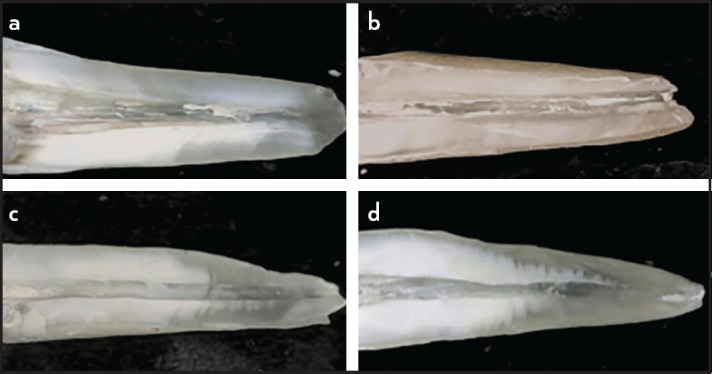
Representative stereomicroscopic photograph 30x showing remaining filling material over root canal surface removed by: (a) G.G+ Hand H file. (b) D-Race, (c) D-Race + XP-Endo Finisher, (d) D-Race + XP- Endo finisher R

As shown in Figure 3 and Table 1, (D-Race + XP-Endo Finisher R) group demonstrated the most significant cleaning efficacy in the mean of residual root canal filling (41.58±10.56), followed by (D-Race+XP-Endo Finisher) group (52.68±9.94); there was no significance with (D-Race) group (55.25±17.71). (G.G+H) group was the least effective in the removal of root canal filling (61.59±15.41), with no significance with (D-Race) group. The efficacy of (D-Race+XP-Endo Finisher R), (D-Race+XP-Endo Finisher) and (D-Race) groups increased apically, while the efficacy of (G.G +H) group increased coronally. Regarding the efficacy among thirds, (G.G+H) group was the most effective in the coronal third. (D-Race+XP-Endo Finisher R) group was the most effective in both middle and apical thirds. 

**TABLE 1.** The mean, standard deviation (SD) and P-values of percentage of the remaining root canal filling materials of different groups in all thirds

Variables	Remaining filling material %								
	Coronal		Middle		Apical		P	Total	
	Mean	SD	Mean	SD	Mean	SD		Mean	SD
Group1	47.21^Cc^	8.83	63.28^Ba^	10.53	74.28^Aa^	12.67	<0.001*	61.59^a^	15.41
(G.G+Hand H-files)									
Group2	70.04^Aa^	10.48	61.58^Ba^	9.04	34.14^Cc^	6.28	<0.001*	55.25^ab^	17.71
(D-Race)									
Group3	56.59^Ab^	8.46	54.78^Aa^	9.19	46.67^Bb^	9.73	0.016*	52.68^b^	9.94
(D-Race and XP finisher)									
Group4	48.17^Abc^	9.03	43.83^Ab^	9.18	32.74^Bc^	7.00	<0.001*	41.58^c^	10.56
(D-Race and XP finisher R)									
P	<0.001*		<0.001*		<0.001*			<0.001*	

*: Indicates significant difference. While different small letters within the same column indicate significant differences among groups, asymmetrical capital letters within the same row indicate significant differences among groups (p<0.05).

Regarding the operating time, (D-Race) group was the fastest with a statistically significance among the tested groups. (D-Race+XP-Endo Finisher R) group demonstrated no statistical significance between groups (D-Race+XP-Endo Finisher) and (D-Race+XP-Endo Finisher R) groups. (G.G+H) group was the slowest one with a statistical significance among them as shown in Table 2.

**TABLE 2.** Total operating time of different groups

Group	Total operating time in minutes
Group1	
(G.G+ Hand H-files)	41.973^A^
Group2	
(D-Race)	26.3206^C^
Group3	
(D-Race and XP-Endo Finisher)	33.3173^B^
Group4	
(D-Race and XP -Endo Finisher R)	32.0977^B^

Indicates significant difference, while asymmetrical capital letters indicate significant differences among groups (p<0.05)

### Debris extrusion

The (G.G+H) group showed the significantly greatest percentage of apically extruded debris (37.49%), followed by the D-Race+XP-Endo Finisher R then the D-Race+XP-Endo Finisher group (16.56 and 12.82%, respectively) (P<0.001). The D-Race group resulted in significantly the least extruded debris (9.12%) (P<0.001) (Table 1).

## DISCUSSION

Regardless of the retreatment technique used, some untouched parts of the root canal filling remain, potentially affecting the prognosis (4). In this study, natural human teeth were used instead of resin blocks, as resin blocks have different features than the natural teeth. Further, the heat generated during root canal preparation and retreatment might soften the resin blocks adhering to the cutting edges (22). Mandibular first premolars were used for standardization purposes because these teeth commonly have type I Vertucci canal configuration, facilitating splitting (22). In the current study, a cold lateral compaction technique was used for obturation using AH Plus sealer as it is the most common technique (15). Several methods have been used for evaluation of the remaining root canal filling after retreatment procedures. 2D radiographic imaging is the most commonly clinical method used (23), in addition to micro-CT (4, 14, 24), scanning electron microscope (25), cone-beam computed tomography (CBCT) (6) and stereomicroscope (26, 27). Stereomicroscope was used to evaluate the remnants of root canal fillings; the device is easy to use, and the object-device distance is constant, enabling image standardization. Besides, the evaluation with qualitative digitalized software that is better than manual qualitative scoring used in other studies (26).

Regarding the remnants of root canal filling after retreatment, in this study, the H files group showed the greatest total amount of remaining root canal filling. Interestingly, H files were the most effective method in the removal of root canal filling from the coronal third. This finding is in accordance with a previous study which could be explained by the large H files used from #60 to #40 (26).

D-Dace 0.04 file was used in the current study because it displayed less amount of filling remnants (27). D Race group was more efficient in removing root canal filling than the H file group in the middle and apical thirds, in accordance with a previous study (26). This is due to the D Race file tip's triangular cross section, high cutting ability, alternating cutting edges, and bullet-shaped design (26). In the coronal third, D-Race was the least efficient; this could be due to the smaller size of files used relative to the H files (with no supplementary approaches) used in groups 3 and 4. Rotary NiTi file with taper similar to that of D Dace/ 0.04 has been investigated in a previous study. It has been showed that the least amount of filling residuals occurred when such file is used without solvents (26).

Although none of the supplementary approaches (XP-Endo Finisher and XP-Endo Finisher R) could completely free the root canals from filling remnants, both significantly decrease the amount of root canal filling. The physical characteristic features of the XP-Endo files, chiefly its ability to transform its shape under the body temperature and expand its action part to 6 mm of its axis, might let the file to touch the root canal walls thus increasing the removal of remnants (28). The activation effect of the XP-Endo files on the irrigation solutions maximizes the cleaning and disinfection of the canal system (26, 28).

**TABLE 3.** The mean and standard deviation (SD) values of apically extruded debris percentage of different groups

Variables	Apically extruded debris %	
	Mean	SD
Group1		
(G.G+ Hand H-files)	37.49%^a^	4.30
Group2		
(D-Race)	9.12%^d^	1.60
Group3		
(D-Race and XP -Endo Finisher)	12.82%^c^	3.41
Group4		
(D-Race and XP- Endo Finisher R)	16.56%^b^	4.07
P	<0.001*	

*: Indicates significant difference, while asymmetrical small letters indicate significant differences among groups (p<0.05)

This finding is in accordance with a previous study (24). It was also shown that the XP-Endo files enabled removal of debris stemming from mechanical preparation, introducing the irrigation solution into the complex anatomies and the untouched walls (29). In the current study, XP- Endo Finisher R was significantly more efficient than XP -Endo Finisher in removing the root canal filling. This could be explained due to larger core diameter and variations in the instruments tip, making it stiffer and more aggressive in retreatment procedures and more efficient in removing root canal filling (3, 6, 27). This result contrasts with a previous study (4). This conflict could be due to different methodology; the previous study used maxillary anterior teeth, root canal instrumentation was operated using Reciproc R25 files, and a different obturation technique was used to achieve a continuous wave of compaction (26). Additionally, XP-Endo Finisher and XP-Endo Finisher R files were used as complementary approaches after Reciproc R25 and R40. Different analytical methods might affect the different results. In this study, a stereomicroscope was used, while in the other study a micro-CT was implemented. Micro-CT scanning generates high-resolution 3D volumetric data that can be used for data processing, quantification, and visualization. This non-destructive technique has been utilized to assess the amount of filling material in the root canal. Additionally, the volume of dentin removed during initial preparation, and the residual filling material and dentine removed after retreatment could be evaluated (4, 14, 24).

Regarding the operating time of retreatment procedures, the findings of our study disclosed that D Race files recorded the least operating time, followed by D Race files with XP-Endo Finisher R, followed by D Race files with XP -Endo Finisher; H file was the slowest. This result could be due to plasticization of gutta-percha from rotary instrumentation, making penetration and retrieval of the softened gutta-percha much easier (21). D Race was the fastest among all groups as it consists of only 2 files with alternating cutting edges which remove the argumentative screwing effect. Furthermore, the smooth surface produced by electro-chemical treatment enabled superior sharpness. Smooth electro-chemicals treated surface of the flutes, increasing the cutting efficiency of D-Race files (21).

Organic and inorganic residues, gutta-percha, sealer, and irrigation solutions might extrude apically throughout the retreatment procedures, resulting in an undesirable consequence. (30). It was shown in a previous study that apical debris extrusion occurred with all instrumentation techniques (7). Regarding the apically extruded debris during retreatment procedures, the results of this study revealed that manual H files recorded the greatest amount of apically extruded debris, followed by D Race files with XP -Endo Finisher R, followed by D Race files with XP -Endo Finisher; D Race files recorded the least amount of apically extruded debris. These results agreed with earlier studies as hand files extruded more debris apically than rotary systems (17, 31). However, an earlier study revealed no significant difference between rotary and hand files in the amount of debris extruded apically (18). But in this study, the rotary file used was not designed for retreatment. D Race system extruded the least amount of debris apically as it consists of only 2 files; as the number of files increases, the apically extruded debris also increases. Additionally, this finding may be attributed to the design of D Race retreatment system adopted in this study. The design has a triangular cross section that minimizes the friction between the file and dentin walls using crown down rotational pressure-less action (17, 30, 32).

XP-Endo Finisher R extruded more debris than XP-Endo Finisher. This could be because XP-Endo Finisher R has a larger core diameter. Additionally, modifications in the instrument’s tip, making it harder and more forceful in retreatment procedures and more efficient in removing root canal filling, increased the debris extruded apically (3).

Since XP-Endo finisher and XP-Endo finisher R are manufactured to be used at high temperatures, the intracanal temperature was simulated in this study. However, the absence of the simulated apical pressure of the periodontal ligament might limit this methodology (15).

The main limitation of this study is that remnants of the filling material after retreatment procedures was not measured using the micro-CT. It is a non-invasive approach that allows for a three-dimensional quantitative evaluation of filling material and dentine removal during retreatment (4, 14, 24), however, it is an expensive experimental tool with limited availability.

Under the conditions of this study, the null hypothesis is rejected. XP- Endo Finisher files seemed to be more effective as supplementary files in the removal of failed gutta-percha after initial removal by D race files. XP- Endo Finisher R files were even more effective than XP- Endo Finisher files. Given the results of the present study, the complete removal of filling material during orthograde root canal retreatment remains a challenge. Thus, the development of new techniques, instruments, or solutions is needed to ensure complete removal of the filling materials from the root canal system. Further studies are needed to investigate the efficacy of XP-Endo Finisher R during retreatment of different obturation techniques using bioceramic sealers. Clinical studies on post-operative pain and flare-up incidence, following the use of XP- Endo Finisher and XP- Endo Finisher R during retreatment procedures, should be also conducted. Further research is also necessary to evaluate the impact of these new supplementary approaches on the outcome of root canal retreatment.

## CONCLUSION

Supplementary techniques are able to reduce the volume of remaining filling materials. XP-Endo Finisher and XP-Endo Finisher R significantly increased the cleaning ability when used as supplementary approaches in endodontic retreatment. However, the XP-Endo Finisher R cleaned canals significantly more than the XP-Endo Finisher and extruded more debris apically. H file was the least effective in cleaning root canals during retreatment. D Race files were the fastest technique in retreatment among all groups.
